# The Role of Insular Cortex and Prefrontal Cortex in the Pathogenesis of Fibromyalgia: Biochemical and Electrophysiological Rodent Study

**DOI:** 10.1007/s11064-025-04617-2

**Published:** 2025-12-11

**Authors:** Ahmed F. Abouelnaga, Abdelaziz M. Hussein, Marwa Abass, Mena Z. Shafiek, Hala F. Zaki, Ahmed F. Mohamed, Weam W. Ibrahim

**Affiliations:** 1 Department of Animal Behaviour and Management, Faculty of Veterinary Medicine, Mansoura University, Mansoura, 35516 Egypt; 2 Department of Medical Physiology, Faculty of Medicine, Mansoura University, Mansoura, 35516 Egypt; 3 Department of Surgery, Anesthesiology, and Radiology, Faculty of Veterinary Medicine, Mansoura University, Mansoura, 35516 Egypt; 4 Postgraduate Program in Pharmacology and Toxicology, Faculty of Pharmacy, Cairo University, Cairo, Egypt; 5 Department of Pharmacology and Toxicology, Faculty of Dentistry, Misr International University, Cairo, 11562 Egypt; 6 Department of Pharmacology and Toxicology, Faculty of Pharmacy, Cairo University, Cairo, 11562 Egypt; 7 Faculty of Pharmacy, King Salman International University (KSIU), South Sinai, 46612 Egypt

**Keywords:** Fibromyalgia (FM), Default mode network (DMN), Deep brain stimulation (DBS), Insular cortex (IC), Medial prefrontal cortex (mPFC), Cingulate gyrus 1 (Cg1), GABA, Glutamate

## Abstract

Neuroimaging implicates the medial prefrontal cortex (mPFC) and insula in fibromyalgia (FM), but synaptic and network mechanisms are unclear. To verify mPFC hypersynapticity in a reserpine-induced FM model and test whether anterior insular deep brain stimulation (DBS) restores mPFC oscillations and pain behavior. Thirty-six male Sprague–Dawley rats received reserpine (1 mg·kg⁻¹ i.p., days 1–3) or vehicle. For Objective 1, mPFC tissue was analyzed by ELISA for glutamate, γ-aminobutyric acid (GABA), C-FOS, nerve growth factor (NGF), synaptophysin, and postsynaptic density protein-95 (PSD-95). For Objective 2, rats were assigned to control-sham, control-DBS, FM-sham, or FM-DBS. Monopolar DBS (130 Hz, 60 µs, 100 µA; 15 min·day⁻¹ for 3 days) targeted the anterior insula; local field potentials were recorded from mPFC, and thermal nociception was assessed by tail immersion and hot-plate tests. Reserpine increased glutamate, C-FOS, NGF, synaptophysin, and PSD-95 and reduced GABA (all *p* < 0.001), confirming hypersynapticity. Insular DBS increased delta-band normalized ratios (NR) in FM and controls (*p* < 0.0001), normalized FM-associated theta reductions, decreased alpha/beta NR in controls, and suppressed elevated gamma NR in FM (*p* < 0.0001). DBS increased withdrawal latencies in FM, indicating improved pain thresholds (*p* < 0.001). Reserpine induces biochemical hypersynapticity in mPFC, and brief anterior insular DBS rebalances mPFC oscillations and alleviates hyperalgesia. Insular DBS may correct cortical network dysfunction in FM.

**Graphical Abstract:**

**Figure:**
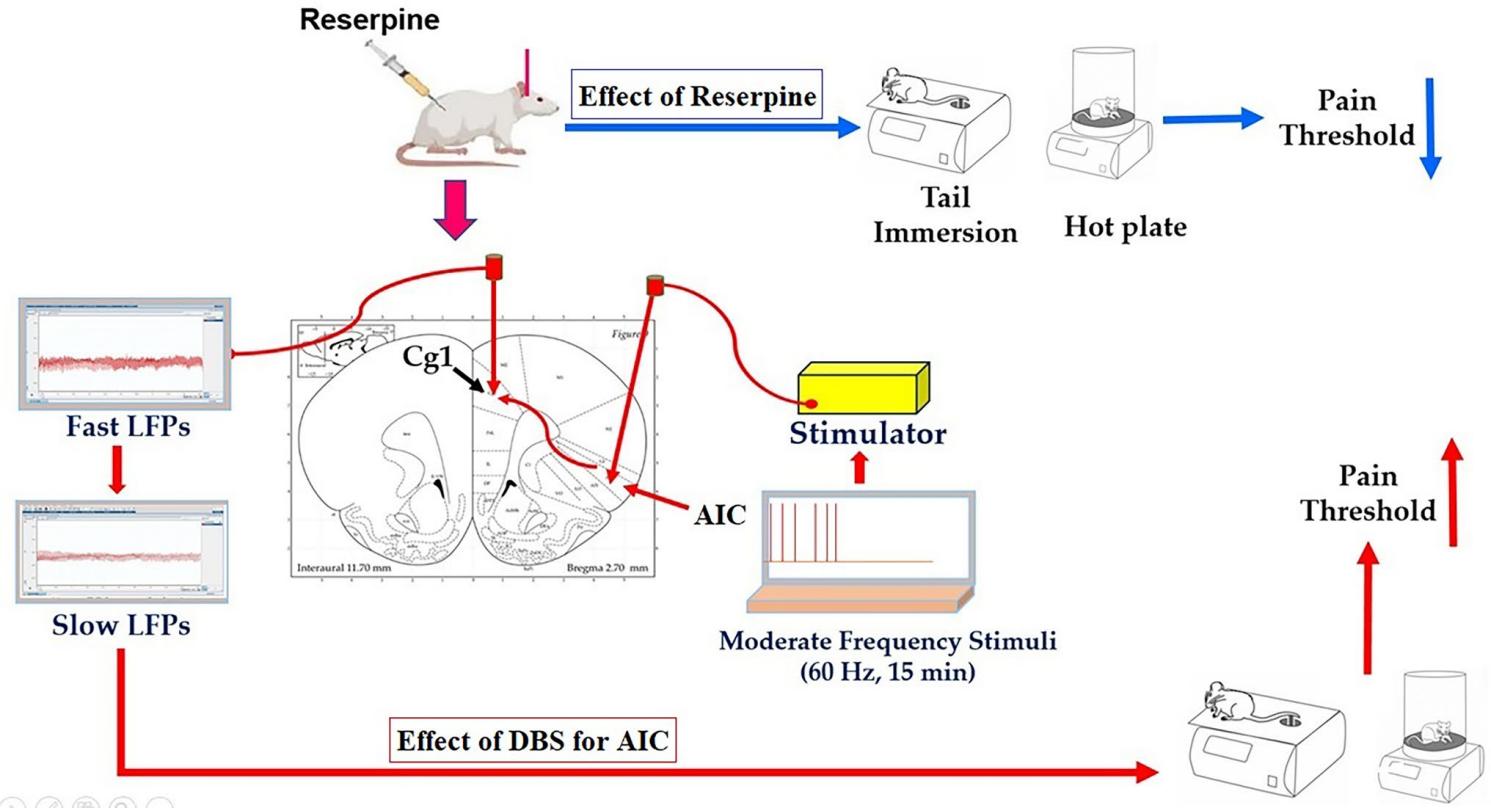
Injection of Reserpine into rats decrease the pain threshold by hot plate and tail immersion tests indicating development of fibromyalgia (FM). On the other hand, deep brain stimulation (DBS) for the anterior insular cortex (dorsal anterior insular cortex, AID) reset the fast rhythms of LFPs into slow LFPs in the cingulate cortex (Cg1) region of the medial prefrontal cortex (mPFC) as well as increase the pain threshold

## Introduction

The medical condition known as fibromyalgia (FM) is characterized by persistent, widespread musculoskeletal pain. This illness’s primary signs include joint and muscle stiffness, erratic sleep habits, exhaustion, mood fluctuations, cognitive decline, anxiety, depression, and difficulty performing daily tasks [1]. Moreover, a variety of disorders, involving diabetes, rheumatic disorders, infections, and neurological or mental health disorders, may be related to FM [2]. The perception of pain is significantly influenced by the insular cortex (IC) and its anterior part (aIC) mainly dorsal angular insular cortex region (AID). Although, the medial prefrontal cortex (mPFC) consists of three regions including infralimbic (IL) and prelimbic (PrL) areas and anterior cingulate gyrus (Cg)1, only Cg1 is considered with nociception. It has previously been shown that FM patients have higher levels of connection between the default mode network (DMN) and insula while they are at rest, and that fluctuations in the degree of this connectivity are linked to variations in the severity of persistent clinical pain [3]. These results indicate that abnormalities in the central nervous system rather than peripheral factors may be the main cause of FM. A compromised mechanism for descending pain inhibition, as well as the inhibition and decreased activity of the brainstem and rostral anterior cingulate gyrus (ACC) (part of medial prefrontal cortex, mPFC), which are brain regions for pain modulation, are some of the potential underlying mechanisms of FM [4, 5].

Pain is characterized as a sensory and emotional experience correlated to an actual or potential tissue injury [6]. Based on its underlying mechanisms, pain can be categorized into nociceptive (physiological) pain and neuropathic (pathological) pain. According to earlier research, neuropathic pain brought on by peripheral nerve damage causes maladaptive alterations in brain regions such as the mPFC [7, 8]. Also, animal model of chronic pain reported functional defects in the mPFC [9]. Additionally, following acute and chronic pain stimuli, the mPFC becomes hyperactive [10]. As pain shifts from an acute to a chronic stage, the mPFC activity decreases [11]. The effectiveness of PFC stimulation in treating patients with neuropathic pain and FM is still unclear, partly because of significant variation in study model [12, 13]. But according to new research in a rat model of chronic constriction injury, repetitive transcranial magnetic stimulation not only inverted thermal hyperalgesia and mechanical allodynia, but it also changed the PFC’s levels of expression of tumor necrosis factor-alpha, brain derived neurotrophic factor, and interleukin 10, which have anti-inflammatory properties. However, the underlying mechanisms, particularly in brain electrophysiology such as local field potential (LFP), are not well known. The LFP is categorized into various frequency bands and is linked to cognitive functions of the brain [14]. It displays low-frequency extracellular activity, including IPSPs and EPSPs [15], and potentials of somato-dendritic spikes [16–18].

Multimodal inputs from many brain regions culminate in the insula, a large cortical region that exhibits differential connection to other brain regions [19]. Additionally, this is reinforced by insular activity during emotional awareness, cardiac awareness, bodily control awareness, and pain perception and modulation [19, 20]. Direct or indirect connections between the anterior IC and spinal cord inhibitory interneurons are made through the mPFC [21]. The perceived intensity of pain is correlated with aIC activation [22], establishing a direct connection between insular brain function and subjective pain perception. Additionally, clinical research highlights how the IC engages in persistent pain [23], demonstrating a correlation between changes in insular activity [24], structure [25], and pain chronification. In pain syndromes, the reason behind pain hypersensitivity is due to enhanced excitability of the insula which is also reinforced by the outcome that FM patients have elevated glutamate amount in the posterior insula, and these levels associate well with pain thresholds [26]. The nociceptive specificity of insular activation is also supported by the findings that thermal nociceptive stimuli cause gamma band oscillation enhancement in the insula [21, 27]. Nonetheless, no study, until now, examined the effect of deep brain stimulation (DBS) for the aIC on the electrophysiological changes in the mPFC in a rat model of chronic nociplastic pain. The insula has a main role in pain processing and is connected to mPFC. Targeting insula with DBS might modulate mPFC activity and pain perception. In FM, it has been demonstrated that there are lower amounts of gamma-aminobutyric acid (GABA) in the insula and posterior cingulate cortex and higher levels of glutamate in these regions. Increased sensitivity to pain in FM has been linked to dysregulated GABA and glutamate balance in areas of the brain that process pain [28]. Glutamate hyperactivity can be caused by either less glutamate reuptake, more glutamate in synaptic vesicles, or a higher number or density of glutamatergic synapses. Given that nerve growth factor (NGF) may play a part in chronic pain and its levels vary in different chronic pain disorders [29, 30], it has been suggested that FM sufferers may exhibit elevated NGF levels, which could exacerbate their pain [31]. Work by Zhang et al. [32, 33] has provided that rats with persistent visceral hypersensitivity have shown indications of purinergic signaling on neurons, with the IC exhibiting an increased level of synaptophysin. Thus, the current study aimed to investigate the role of anterior IC (aIC) and mPFC (Cg1) in the pathophysiology of FM by proposing two objectives. The first objective was to verify the presence of hypersynapticity in the mPFC of rats with reserpine-induced FM *via* biochemical assay of mPFC levels of neurotransmitters. Upon confirmation, the second objective was to determine whether short-course DBS of the aIC can normalize mPFC (Cg1) electrophysiological oscillations and improve pain-related behavioral outcomes in the same model.

## Materials and Methods

### Animals

We acquired 36 male Sprague Dawley rats, weighing 200–250 g, from the animal house at Cairo University’s Faculty of Pharmacy in Cairo, Egypt (twelve rats) and Department of Medical Physiology, Mansoura Faculty of Medicine, Egypt (Twenty-four rats). The animals were housed in controlled environments with 22 ± 2 °C temperatures, humidity levels between 50 and 70%, and a 12-hour cycle of light and dark, with lights shutting off at 7:00 AM, throughout the experiment. Sawdust was used as bedding material as a standard housing requirement in all cages. Male rats were selected for the study to eliminate the potential influence of female estrous cycles on behavioral and physiological outcomes. They received an accommodation period one week before the testing procedures, during which they were provided with unlimited access to water and rat food.

### Compliance with Ethical Standards

The study was approved by the Faculty of Pharmacy’s Ethics Committee on Animal Care and Use, Cairo University, under Permit number 3458. Every investigative method was conducted in compliance with the guidelines established by the US National Institutes of Health’s Guide for the Care and Use of Laboratory Animals (NIH publication No. 85-23, revised 2011) and the ARRIVE guidelines. All attempts were made to keep the animals as comfortable as possible during study.

### Drugs and Chemicals

From Sigma-Aldrich Chemical Co. (St. Louis, MO, USA), reserpine was bought, and it was dissolved in distilled water with 0.5% acetic acid. All compounds were of the purest and best analytical quality.

### Experimental Design

#### Experiment # 1

##### Study Design

For the first objective, twelve rats were chosen to verify the presence of hypersynapticity in the mPFC and these animals were randomly allocated into 2 groups (each 6 rats) as follows:

i) **Control group**: rats were subcutaneously injected with 0.5% acetic acid in distilled water for 3 consecutive days.
ii) **FM group**: rats were administered reserpine (1 mg/kg/day) for 3 consecutive days [34, 35].

##### Animal Sacrifice and Brain Harvesting

At the end of experiment, under phenobarbital anesthesia (40 mg/kg; intraperitoneally), rats were euthanized [36], then the brain was perfused with 150 ml saline via cardiac catheterization, after that the brain was isolated, rinsed with saline, dried, and weighed [37, 38]. After being frozen in liquid nitrogen, the samples were kept at −80 °C for biochemical analyses of neurotransmitters levels in mPFC.

##### ELISA Assay for Biochemical Parameters

The mPFC was dissected from the brain tissues, then homogenized in PBS using Pestle and mortar. In tissue homogenate, using ELISA kits supplied by Mybiosource (CA, USA), the concentrations of glutamate (Cat# MBS756400), GABA (Cat# MBS269152), C-FOS (Cat# MBS729725), NGF (Cat# MBS261790), and synaptophysin (Cat# MBS702560) were measured by adhering to the instructions given by the pertinent kit. Additionally, post synaptic density 95 (PSD-95) was quantified by Lsbio, (TX, USA) ELISA kit (Cat# LS-F7142). Tissue homogenates’ protein content was assessed using the technique of [39]. The experimenter was blind to the rat’s group.

#### Experiment #2

##### Study Design

For the second objective (examining the effect of DBS for the IC on the electrophysiological and pain threshold changes in the mPFC), twenty-four rats were divided haphazardly into 4 groups (each 6 rats) as follows:

1. **Control without DBS group**: rats underwent a sham surgical operation that involved the placement of recording and stimulating electrodes on the right hemisphere of the brain, without applying DBS. Then received a subcutaneous injection of 0.5% acetic acid in distilled water for a period of 3 days.
2. **Control with DBS group**: rats underwent surgical implantation of recording and stimulating electrodes on the right side of the brain, then received a subcutaneous injection of 0.5% acetic acid in distilled water for a period of 3 days before DBS. These electrodes enable the delivery of DBS to the insula while simultaneously recording mPFC activity.
3. **FM without DBS group**: rats underwent surgical implantation of recording and stimulating electrodes on the right side of the brain, without DBS, and were administered reserpine (1 mg/kg/day) after recovery for 3 consecutive days [34, 35, 40].
4. **FM with DBS group**: rats exposed to surgical implantation of recording and stimulating electrodes on the right side of the brain with DBS and were administered reserpine (1 mg/kg/day) after recovery from surgical implantation for 3 consecutive days before DBS.

##### Surgery and Implantation of the Electrodes

Rats were anesthetized by intraperitoneal (IP) administration of 0.4 mg/kg medetomidine (Domitor 1, Pfizer, Seixal, Portugal) and 50 mg/kg ketamine (Ketamax 5%, Troikaa, Gujarat, India) [41]. Both corneas were treated with eye cream to prevent corneal ulcers. A heating pad was used to maintain rats’ body temperatures in the range of 37.5 to 38.5 °C. We leveled the rat’s skull between the bregma and lambda and fastened it in the digital stereotaxic frame device (model # 68025, RWD life sciences, China) during the procedure. A unilateral bipolar stimulating electrode (#MS303/1-B/SPC twisted stainless steel, outer diameter 250 μm, Plastics One, USA) was then implanted in the ***anterior Insula*** (dorsoventral: − 6.5 mm from skull surface, mediolateral: + 4.5 mm, and anteroposterior: + 1.5 mm). To provide sufficient room for the recording electrode to be inserted, electrodes for stimulation were inserted at a 9º angle. We next implanted an ipsilateral recording electrode (MS333/1-C/SPC stainless steel twisted insulated three channels’ electrodes, outer diameter 250 μm, Plastics One, USA) in the ***mPFC (anterior Cingulate gyrus)*** (dorsoventral: − 2.5 mm from skull surface at 0 angle, mediolateral: + 0.3 mm, and anteroposterior: + 2.5 mm). Three screws and dental cement were utilized to attach electrodes to the skull. Both recording and stimulating electrodes were implanted on the right side of the brain. After surgery, animals were intensively monitored for seven days. The study eliminated animals exhibiting any neurological symptoms of brain injury.

##### Protocol of DBS for the Anterior Insula

DBS was carried out by using high-frequency (HF) electric biphasic square pulses (100 µA amplitude, pulse width of 210 µS, and frequency of 60 Hz) for 15 min over a period of 3 consecutive days after FM induction by reserpine. Using a stimulus isolator (FE180, ADInstruments, USA), stimuli were given [42, 43].

##### In Vivo Electrophysiological Recordings of LFPs from mPFC

The ML136 animal Bio Amplifier (ADInstruments, USA) and Powerlab 3/40 data acquisition system were used for electrophysiological recording at the Department of Medical Physiology, Mansoura Faculty of Medicine. The LFPs from mPFC were recorded in awake rat, 5 min after stimulation [44] under the following criteria (50 Hz notch filter, main filters, electroencephalogram (EEG) mode, sampling rate 1k/s., range: 1 mv or less). Power spectra are produced by using the Fast Fourier Transform analysis method to the LFP recordings, which converts data from the time domain to the frequency domain. The obtained averaged power spectra were used to identify five frequency bands: delta (0.1–4 Hz), theta (4.1–8 Hz), alpha (8–14 Hz), beta (14–30 Hz), and gamma (30–50 Hz) [45]. Normalizing band power was accomplished by dividing the value of each animal’s individual band power by the total power of all bands, then multiplying the result by 100 to obtain a ratio for each band. This allowed for comparison and helped to overcome inter-individual differences. Normalization ratio (NR) = (power of each band/total power of all bands) × 100.

##### Behavioral Tests

Tail Immersion Test: Assessing Mice’ Spinal Heat Sensitivity Is the Aim of the Tail Immersion Test. The Experimenter Was Blind To the Rat’s group. After Submerging the Distal 1 Cm Section of the Tail in a Water Bath Kept at a Steady Temperature of 55 °C, the Nociceptive Reaction time, Expressed in seconds, Was determined. A Quick Flinch of the Rat’s Entire Body or a Violent Shake of Its Tail Were Used To Identify the Nociceptive endpoint. A 15-second Maximum cut-off Period Was Established To Avoid Tissue Damage [46]. We Started with the Tail Immersion Test before Hotplate Test because It Is Less Stressful for the Rats.

Hot Plate Test: the Hot Plate Test Is Used To Measure Thermal Hyperalgesia and Is One of the Most Popular Techniques for Assessing Supraspinal Thermal Nociception in rodents. the Experimenter Was Blind To the Rat’s group. In this study, a Hot Plate “Ugo Basile, Italy, Model 7280” Kept at a Temperature of 54–56 °C Was Used. Rats Were Put in, and the Thermal Pain Behavior Latency Was Measured by How long It Took Them To Lick their Hind Paw or Jump Out of the cage. To Avoid Possible Physical injury, a 12-second Reaction time Cutoff Was Set [47].

#### Tracking of Stimulating and Recording Electrodes in Target Regions

**Fig. 1:**
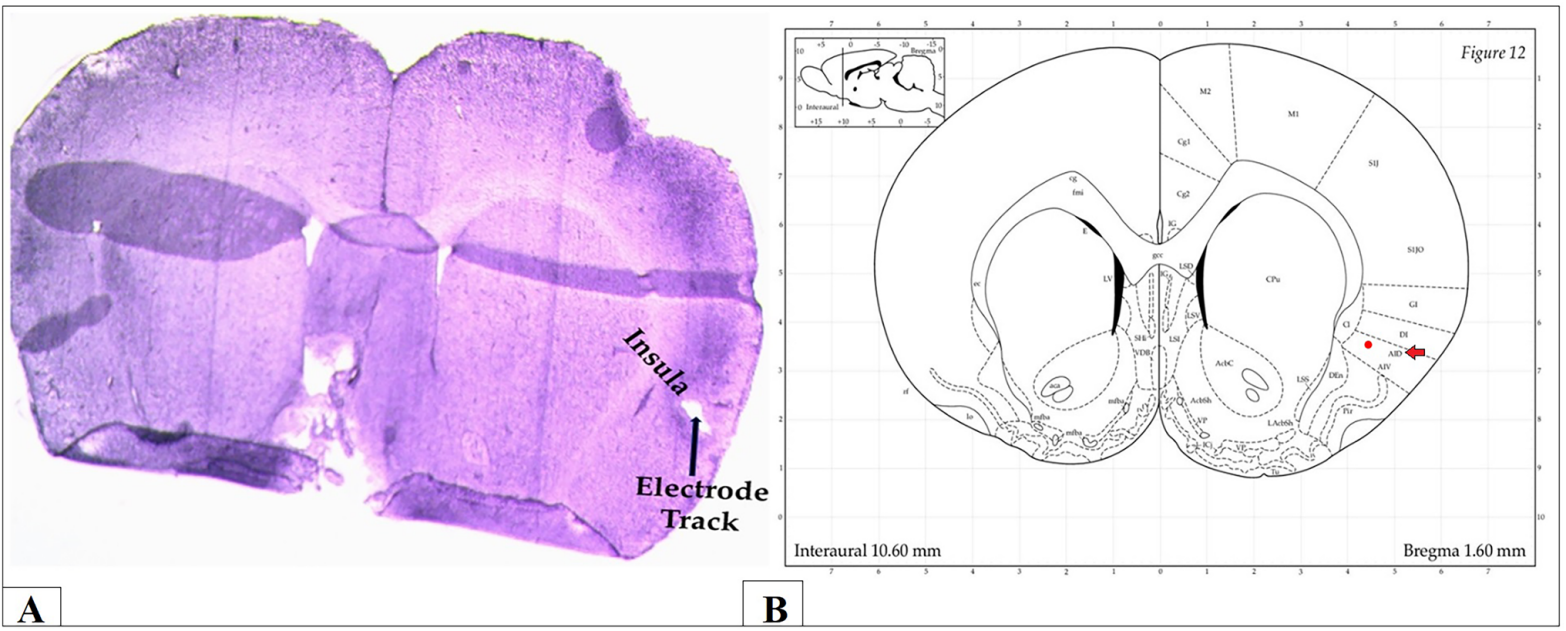
Tracking of the stimulating electrodes. **A** Anterior insular cortex (AI) in brain sections obtained from rats and **B** Atlas of brain showing the anterior insular cortex (dorsal anterior agranular cortex, AID) (red arrow) in coronal section of brain

**Fig. 2:**
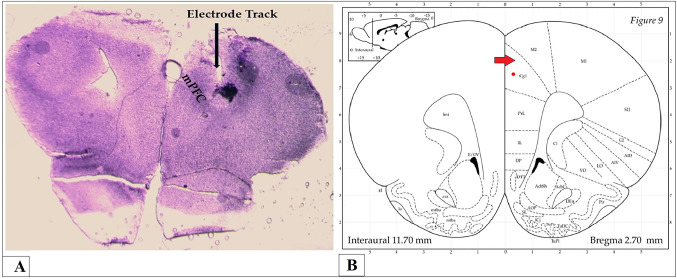
Tracking of the recording electrodes. **A** mPFC or anterior cingulate gyrus in brain sections obtained from rats and **B** Atlas of brain showing the anterior cingulate cortex or gyrus (Cg1) (red arrow) in coronal section of brain

Behavioral tests were conducted for both control and FM groups prior to and after DBS, with a two-hour resting period between each set of assessments. After completing the behavioral tests, under phenobarbital anesthesia (40 mg/kg; intraperitoneally), rats were euthanized [36]. The brains of rats were harvested via cardiac perfusion with 150 ml formaldehyde (10%), fixed in formalin (10%), and then placed in 30% sucrose solution for 48 h [37, 38]. After that, brains were fixed into O.C.T. gel (Tissue-Teck) for obtaining cryosections (20 μm) using cryostat for tracking the stimulating electrodes in insula and the recording electrodes in mPFC. Figure 1A, B show the track of stimulating electrodes in the anterior insula (aIC) specifically in dorsal agranular insular region (AID), while Fig. 2A, B show the recording electrodes in mPFC (Cg1 region).

### Statistical Analysis

Statistical analyses were done using graphPrism version 6.0. For detection of the normality of the data Shapiro-Wilk test was used. For detection of statistical significance between 2 groups, independent T test was used, while one-way ANOVA with the Bonferroni post hoc test was utilized to find the statistical significance among different groups. The mean ± standard deviation of the mean is used to display all the data.

## Results

### Effect of Reserpine Administration on Synaptic Biomarkers, GABA and Glutamate Balance in mPFC in FM Rats

Reserpine-injected rats showed synaptic dysfunction as indicated by a marked rise in mPFC levels of NGF (2.96 folds), PSD-95 (2.85 folds), synaptophysin (2.24 folds), and C-FOS (3.2 folds) as compared to the control group. This is accompanied by dysregulated GABA and glutamate balance presenting marked decline in GABA levels (56.56%) contrary to elevated glutamate levels by 3.33-folds as compared to the control group (Table 1).

**Table 1:** Represents the effect of reserpine administration on synaptic biomarkers, GABA and glutamate balance in mPFC in FM rats

Parameter						
Group	Glutamate (pg/mg protein)	GABA (pg/mg protein)	NGF (pg/mg protein)	PSD-95 (ng/mg protein)	Synaptophysin (ng/mg protein)	C-FOS (ng/mg protein)
Saline control group	48.77 ± 2.757	124.3 ± 4.314	116.9 ± 3.18	3.417 ± 0.3357	30.57 ± 3.411	3.04 ± 0.3134
FM group	162.3 ± 19.91	70.3 ± 2.9	346.6 ± 18.91	9.733 ± 1.266	68.53 ± 7.337	9.733 ± 0.5241
Percentage Change	3.33 folds	56.56%	2.96 folds	2.85 folds	2.24 folds	3.2 folds
*p* value (Independent T test)	*P* < 0.0001	*P* < 0.0001	*P* < 0.0001	*P* < 0.0001	*P* < 0.0001	*P* < 0.0001

Data are expressed mean ± SD. Independent T test

### Effect of DBS for IC on LFPs in the mPFC Region in Reserpine-Induced FM Rats

**Fig. 3:**
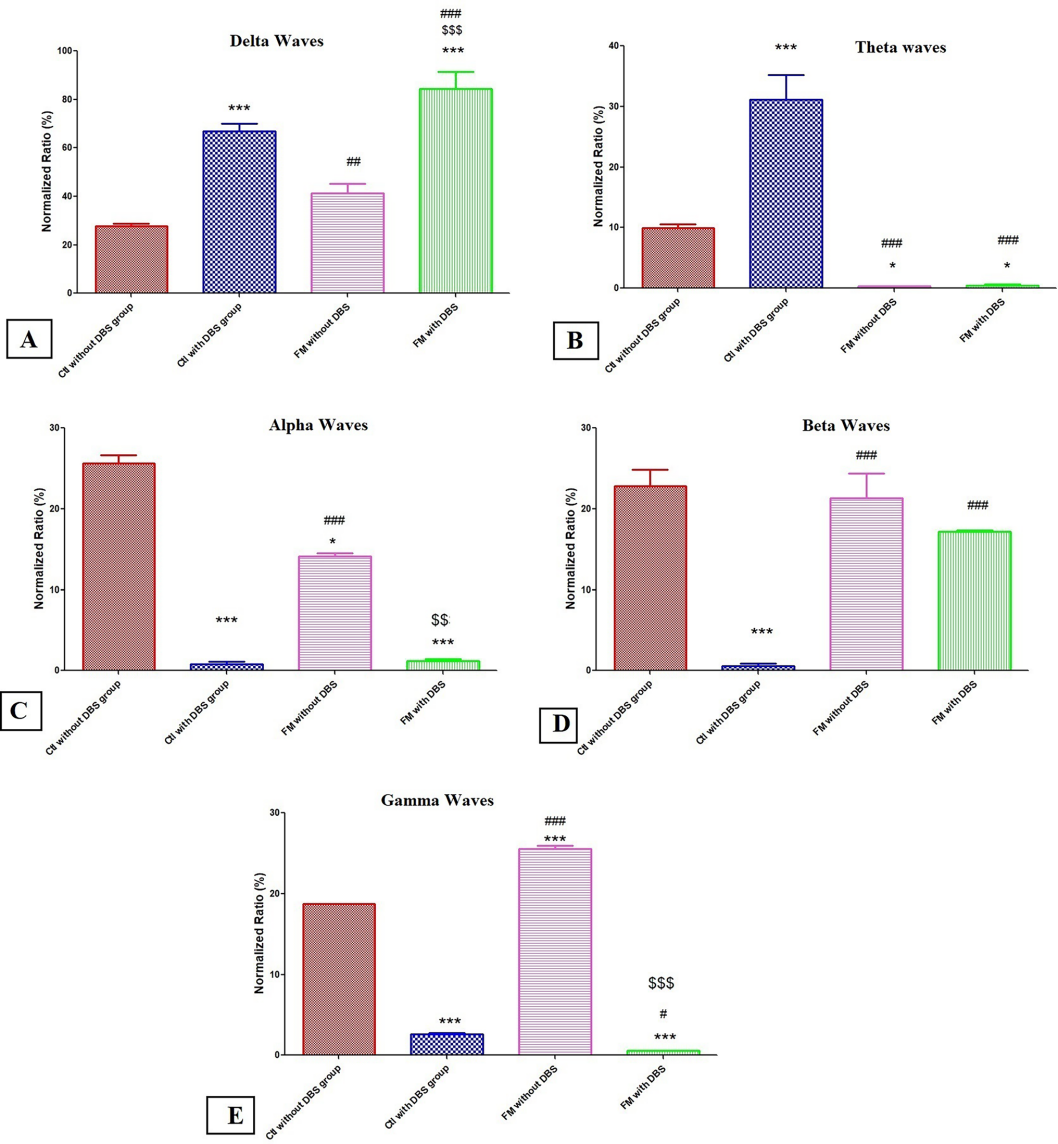
Normalized ratios (NR) of local field potentials (LFPs) from different groups. **A** NR of delta waves, **B** NR of theta waves, **C** NR of alpha, **D** NR of beta waves and **E** NR of gamma waves. ^*^ vs. control without DBS, ^#^ vs. control with DBS and ^$^ vs. FM without DBS group; * *p* < 0.01, ** *p* < 0.001 and *** *p* < 0.0001. Each bar with vertical line represents mean ± S.D. (*n* = 24) using one-way ANOVA with the Bonferroni post hoc test; *p* *<* 0.05

**Fig. 4:**
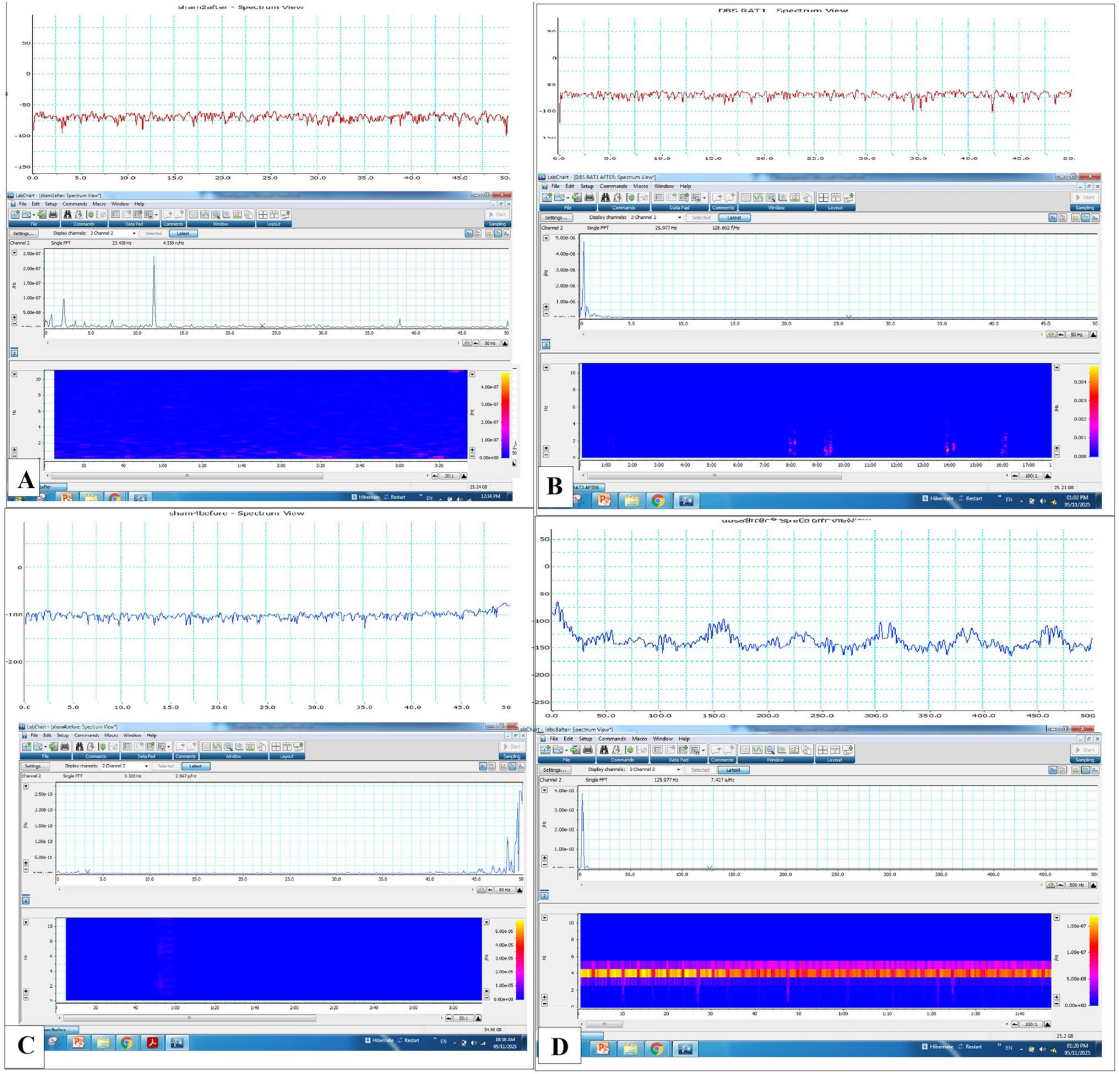
Raw records and power spectra of local field potentials (LFPs) from **A** control without DBS group, **B** control with DBS group, **C** FM without DBS group and **D** FM with DBS group

Both control and FM groups with DBS had significantly greater NR of delta waves of LFPs recorded from mPFC than their respective groups (*p < 0.0001*) (Fig. 3A). Concerning the theta waves, DBS considerably raised their NR in control rats exerting null effect in FM group, noting that the NR of theta waves were lower in the FM groups than in their comparable control groups (*p < 0.0001*) (Fig. 3B). The NR of alpha waves was significantly decreased in the FM group than in the control group (*p < 0.0001*) and the NR of gamma waves was statistically markedly elevated in the FM group as compared to the control group (*p < 0.0001*) and DBS considerably reduced the NR of alpha and gamma waves in both control and FM groups as compared to their respective groups (*p < 0.0001*) (Fig. 3C, E). Regarding the beta waves NR, there was no statistically significant differences among the study groups, except for the Ctl with DBS group which presented a much lower NR than the other groups (Fig. 3D). Figure 4A–D shows raw records and power spectra of LFPs recorded from different groups.

### Effect of DBS on Pain Threshold in Reserpine-Induced FM Rats

**Fig. 5:**
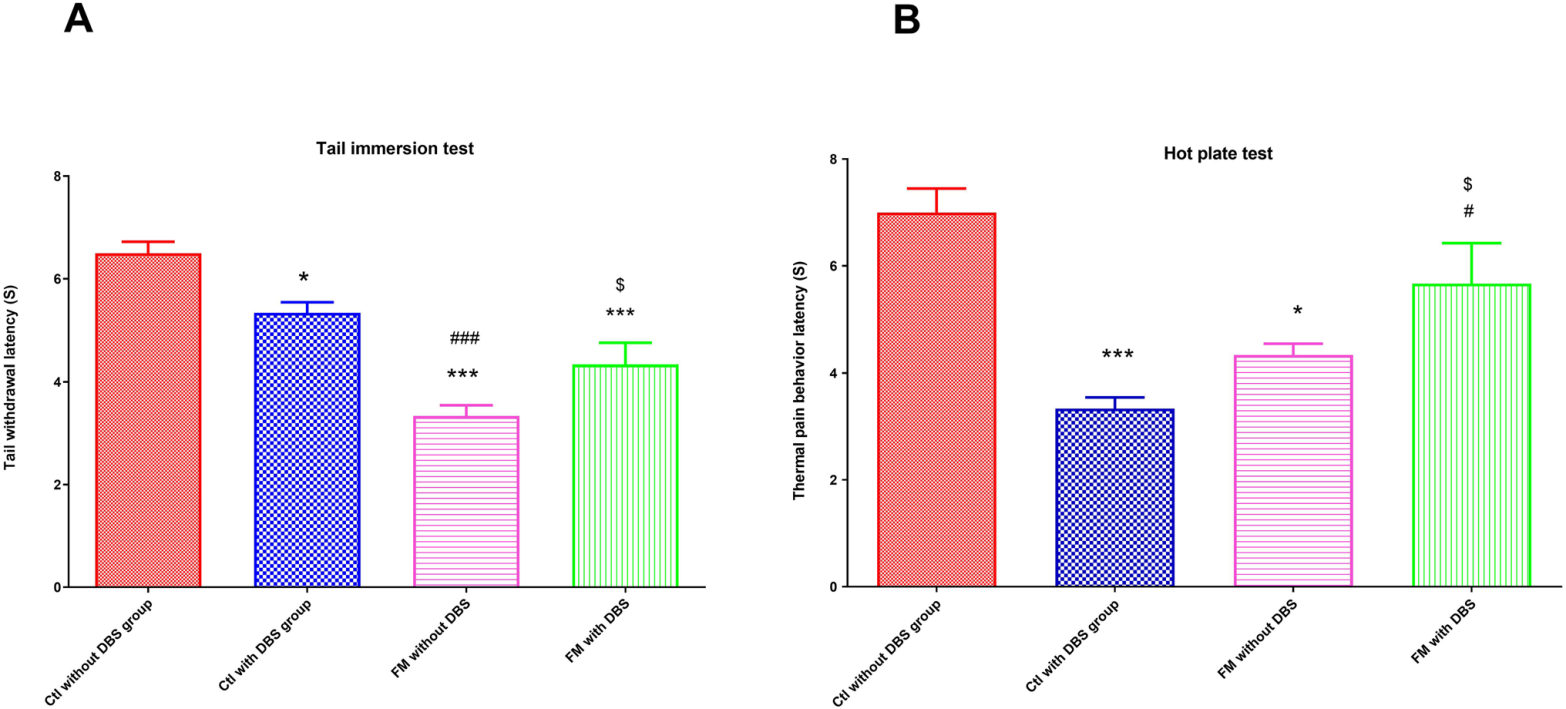
Pain sensitivity threshold from different groups assessed by **A** tail immersion test and **B** hotplate test. ^*^ vs. control without DBS, ^#^ vs. control with DBS and ^$^ vs. FM without DBS group; * *p* < 0.01, ** *p* < 0.001 and *** *p* < 0.0001 Each bar with vertical line represents mean ± S.D. (*n* = 24) using one-way ANOVA with the Bonferroni post hoc test; *p* *<* 0.05

FM without DBS group showed significant reduction in pain threshold as witnessed in tail immersion (*p < 0.0001*) and hot plate test (*p < 0.01*) compared to the corresponding control group. Reserpine-induced hyperalgesia and allodynia was ameliorated by DBS which displayed a marked increase in values of hot plate and tail immersion tests as compared to FM without DBS group (*p < 0.001*). Also, pain threshold was significantly decreased in control group with DBS as compared to control group without DBS (*p < 0.001*) (Figs. 4B and 5A).

## Discussion

The present study verified the existence of hypersynapticity in reserpine-induced FM rats, particularly in the mPFC, a brain region intimately involved in pain modulation. This was evidenced by a substantial increase in mPFC contents of NGF, synaptophysin, PSD-95, and C-FOS in FM group rats. Persistent neuropathic pain may cause the mPFC to express more C-FOS [48]. Additionally, it was demonstrated that spinal neurons display a notable increase in C-FOS after a nociceptive stimulus [49]. The spinal cord’s pain pathway has noticeably more asymmetric synapses than symmetrical ones [50]. PSD95 is a postsynaptic scaffolding protein of excitatory synapses, and its levels are increased during pain production. It is essential for synapse stability and plasticity, and it encourages synaptic development and remodeling [51–54]. It has been reported that the increase in PSD95 expression was accompanied by an increase in dendritic spines [51]. In the rat cortex, PSD95 thickness and expression are increased in response to neuroinflammatory diseases and brain injury [55] in accordance with the present results. Moreover, excessive NMDA receptor activation brought on by the PSD95-neuronal nitric oxide synthase complex results in pathological pain [56]. The increased levels of NGF, reported herein, produces hypersensitivity and encourages neuronal growth and synaptic remodeling. Likewise, the elevated synaptophysin and PSD-95 indicates possible synaptic overgrowth or heightened synaptic function that leads to an increase in transmission of pain mediators.

According to our findings, a drop in GABA levels (inhibitory neurotransmitter) is thought to be the source of persistent pain [57]. Although the cause of FM is still not fully understood, there is mounting evidence that people with FM have dysregulated pain processing in their central nervous systems, which is particularly connected to high levels of the glutamate (excitatory neurotransmitter) in the brain. Furthermore, there is evidence linking increased glutamate levels to an increase in symptoms of FM [58]. This notion is supported by decrease in time latency in hot plate test and withdrawal latency in tail immersion test in FM rats [35, 40]. In cortical circuits, the increase in glutamate and decline in GABA predispose neurons to fire at higher rates and more synchronous bursts [59]. This aligns well with elevated gamma power observed in FM rats. Gamma oscillations reflect synchronized high-frequency firing and excessive gamma is often linked to hyperexcitability or hypersensitivity to neurons.

FM is a chronic pain condition whose cause is still unknown. Studies using functional magnetic resonance imaging (fMRI) on FM subjects have revealed changed connections between the DMN and the insula [60]. FM patients have elevated brain activities in the so-called “pain network,” which includes the thalamus, ACC, primary (S1) and secondary somatosensory cortices, and IC. Additionally, both painful and nonpainful stimuli cause them to become hypersensitive [61, 62]. fMRI studies have shown that the brain connection to this pain network has altered in FM, and certain intrinsic connectivity networks have shown synchronous activity while at rest (i.e., resting-state networks), such as the DMN [63, 64], salience network [3], and sensorimotor network [65]. Also, previous animal studies demonstrated reciprocal connections between anterior cingulate cortex (ACC) and anterior insula (AI) which is important in gating the nociceptive thalamic input to ACC/medial prefrontal cortex (mPFC) [66, 67]. Generally, the persistent and ongoing pain experienced by FM patients seems to affect how their brains respond to information from the external environment and internal thinking. The DMN consists of a number of synchronous brain regions, like the mPFC and posterior cingulate cortices, which are inactive during task performance and active during rest [68]. Because the insula-DMN link can objectively diagnose FM symptoms and transmit clinical pain, more research on this relationship is necessary. The perception of pain is greatly influenced by the DMN and insula, which might display related behaviors in lots of tasks, such as paying attention [69, 70]. It has been proposed that during pain processing, the insula serves as a switching center, transforming sensory data into more complex emotional and mental control [71, 72]. Luckily, the DMN’s capacity to regulate pain has been connected to descending inhibitory networks [73]. Consequently, the current discovery of impaired insula-DMN connectivity may be related to poor control of pain, which ultimately results in FM’s chronic pain. Similarly, MRI studies in FM revealed that DMN regions displayed decreased gray matter volume [74] and a functional link to specific pain network regions [75]. One of the conflicting results in the current study was that DBS decrease pain threshold in control rats but increase pain threshold in FM rats. This can be explained on the bases of DBS doesn’t have a “one-size-fits-all” effect — it modulates rather than simply “activates” or “inhibits” brain regions and its net effect depends heavily on the baseline excitability and synaptic tone of the network being stimulated. The aIC in healthy rats already has a balanced excitatory–inhibitory tone. So, DBS to aIC in healthy rats can disrupt this balance, leading to increased stress or aversive affect (since the insula contributes to emotional salience of pain), enhanced attention to nociceptive input and dysregulated descending modulation (from ACC, amygdala, and brainstem nuclei like periaqueductal grey area (PAG) and RVM), so pain threshold decreases i.e., rats become more sensitive to pain. On the other hand, in FM models there’s dysfunctional connectivity in the aIC, blunted descending pain inhibition (PAG pathway) and neurotransmitter imbalance (↓ serotonin, ↓ dopamine, ↑ glutamate) causes chronic hyperalgesia. So, when DBS is applied to the aIC in case of FM restores the disrupted excitatory–inhibitory balance, enhances descending pain inhibition (recruiting PAG circuits) and normalizes insular–limbic communication, reducing emotional amplification of pain. So, pain threshold increases i.e., the animals become less sensitive to pain.

The extracellular records of neuronal membrane potentials including EEG or local field potentials (LFPs) reflect several mental processes. It has been demonstrated that the theta wave oscillations are linked to working memory, attention, emotional arousal, and fear conditioning, while beta oscillations are linked to top-down attention regulation in pain processing [76] and the perceptual integration of sensory (cognitive, emotional, and motivational) and contextual information [77, 78]. On the other hand, gamma wave oscillations have been linked to a variety of behavioral situations, including movement, memory, attention, decision-making, anxiety and movement [79]. Also, previous studies demonstrated that gamma waves reflect the hypersynapticity states and imbalance between GABA/Glutamate and the use of a GABA-A receptor antagonis (PTZ) interfere with regular brain wave patterns with an increase in gamma oscillations [80, 81]. Regarding the electrophysiological changes in EEG waves and LFPs waves in patients with chronic pain, the different studies in scientific literature demonstrated controversial findings. Ploner et al. reported that the theta oscillation is the chief alteration in brain rhythm occurred in patients with chronic pain [78]. Also, the resting EEG and MEG in FM have found changed theta oscillations in the mPFC and other midline brain areas [82, 83]. Sarnthein et al. and Stern et al. found that people with persistent neuropathic pain had higher resting EEG power in the theta and beta frequency bands localized to the anterior cingulate, prefrontal, and somatosensory cortices [84–86]. Moreover, the patients with migraine and complicated regional pain syndrome have also been found to exhibit increases in low-frequency spectral power in their resting brain EEG [87, 88] and in mood disorders, oscillation has been proposed as the neuropathological mark of brain rhythm [89], cognitive impairment [90], pain attack [91], and fatigue [92].

The current study found that rats with FM had low theta and alpha with high gamma frequency bands in mPFC region compared to normal rats. But there is no statistically significant difference between normal rats and FM rats in the current study in the other waves (beta and delta waves). On the other hand, DBS of the insular cortex caused significant increase in delta waves in both normal and FM rats and theta waves in normal rats not FM rats. Moreover, DBS caused significant decrease in gamma rhythms in normal and FM rats and significant decrease in alpha and beta waves in only normal rats as compared to their corresponding groups. The findings of the current study are not in agreement with the previous studies in the scientific literature except the delta waves (low frequent waves) and gamma waves (high frequent waves) in case of FM compared to normal control group and DBS for the insula significantly reduced the high frequent gamma wave and increased the low frequent delta waves suggesting that DBS for the IC could reset the frequency of mPFC frequency waves by reducing the high frequency waves and increasing low waves. In line with these findings, Liberati et al. demonstrated that thermal nociceptive stimuli cause gamma band oscillation enhancement in the insula [21, 27]. Although, the electrophysiological changes in Cg1 region of mPFC after deep stimulation for the aIC is the first study, up to the best of our knowledge, the results of LFP in the present studies are just primary findings in animal studies which need further clarifications and confirmations using high resolution techniques such as optogenetics and fiber photometry which are more specific for the brain regions and cell types. Also, deep analyses for LFPs such as coherence and cross frequency coupling which needs specific softwares such as MATLAB which is not available in our lab is one of the limitations that will be considered in future studies. Moreover, most of the findings in previous studies come from EEG recordings in human studies not LFPs from specific brain regions in animal studies and this could explain the controversy in the findings between the current study and previous studies. Moreover, the place of recording of LFPs and the effect of DBS depends upon the frequency, amplitude of the current used as well as the duration of stimulation could be factors that influence the modality of the wave recorded.

Another limitation of our study is the inability to assess the effects of DBS on the structural and neurochemical alterations associated with FM in the mPFC. This was due to the processing of brain tissue for electrode localization, which involved fixation in 10% formalin, cryoprotection in 30% sucrose, and embedding in O.C.T. compound for cryosectioning. As a result, further studies are recommended (using optogenetic techniques in transgenic animals or transcranial magnetic stimulation (TMS) in human) to specifically evaluate DBS-induced changes in neuronal integrity and neurotransmitter profiles in the mPFC.

## Conclusion

The current study sheds light on the ameliorating effects of DBS on FM model. It paints a cohesive picture of how chronic pain states in FM are sustained by altered network connectivity within the mPFC-insula circuit by elevating the delta band and suppressing alpha, gamma, theta, and beta bands in addition to counteracting the excitatory/inhibitory imbalance by altering network dynamics.

## Data Availability

No datasets were generated or analysed during the current study.

## References

[1] Siracusa R, Di Paola R, Cuzzocrea S, Impellizzeri D (2021) Fibromyalgia, pathogenesis, mechanisms, diagnosis and treatment options update. Int J Mol Sci 22:3891 33918736 10.3390/ijms22083891 PMC8068842

[2] Bellato E, Marini E, Castoldi F, Barbasetti N, Mattei L, Bonasia DE et al (2012) Fibromyalgia syndrome: etiology, pathogenesis, diagnosis, and treatment. Pain Res Treat. 10.1155/2012/426130 23213512 10.1155/2012/426130 PMC3503476

[3] Ichesco E, Schmidt-Wilcke T, Bhavsar R, Clauw DJ, Peltier SJ, Kim J et al (2014) Altered resting state connectivity of the insular cortex in individuals with fibromyalgia. J Pain Churchill Livingstone 15:815-826.e1 10.1016/j.jpain.2014.04.007 PMC4127388 24815079

[4] Sawaddiruk P, Paiboonworachat S, Chattipakorn N, Chattipakorn SC (2017) Alterations of brain activity in fibromyalgia patients. J Clin Neurosci Churchill Livingstone 38:13–22 10.1016/j.jocn.2016.12.014 28087191

[5] Yunus MB (2007) Fibromyalgia and overlapping disorders: the unifying concept of central sensitivity syndromes. Semin Arthritis Rheum 36:339–356 17350675 10.1016/j.semarthrit.2006.12.009

[6] Brietzke AP, Zortea M, Carvalho F, Sanches PRS, Silva DPJ, Torres IL da (2020) Large treatment effect with extended Home-Based transcranial direct current stimulation over dorsolateral prefrontal cortex in fibromyalgia: A proof of concept Sham-Randomized clinical study. J Pain Churchill Livingstone 21:212–224 10.1016/j.jpain.2019.06.013 31356985

[7] Thompson SJ, Millecamps M, Aliaga A, Seminowicz DA, Low LA, Bedell BJ et al (2014) Metabolic brain activity suggestive of persistent pain in a rat model of neuropathic pain. Neuroimage Acad Press 91:344–352 10.1016/j.neuroimage.2014.01.020 PMC3965588 24462776

[8] Hung KL, Wang SJ, Wang YC, Chiang TR, Wang CC (2014) Upregulation of presynaptic proteins and protein kinases associated with enhanced glutamate release from axonal terminals (synaptosomes) of the medial prefrontal cortex in rats with neuropathic pain. Pain 155:377–387 24211726 10.1016/j.pain.2013.10.026

[9] Metz AE, Yau HJ, Centeno MV, Apkarian AV, Martina M (2009) Morphological and functional reorganization of rat medial prefrontal cortex in neuropathic pain. Proc Natl Acad Sci 106:2423–2428 19171885 10.1073/pnas.0809897106 PMC2650172

[10] Lin HC, Huang YH, Chao THH, Lin WY, Sun WZ, Yen CT (2014) Gabapentin reverses central hypersensitivity and suppresses medial prefrontal cortical glucose metabolism in rats with neuropathic pain. Mol Pain 10:1–12 25253440 10.1186/1744-8069-10-63 PMC4182821

[11] Buzsáki G, Draguhn A (2004) Neuronal oscillations in cortical networks. Science 304:1926–1929 15218136 10.1126/science.1099745

[12] O’Connell NE, Marston L, Spencer S, Desouza LH (2018) Wand BM Non-invasive brain stimulation techniques for chronic pain. Cochrane Database Syst Rev. 10.1002/14651858.CD008208.pub4 29547226 10.1002/14651858.CD008208.pub4 PMC7039253

[13] Knotkova H, Hamani C, Sivanesan E, Le Beuffe MFE, Moon JY (2021) Cohen SP Neuromodulation for chronic pain. Lancet 397:2111–2124 34062145 10.1016/S0140-6736(21)00794-7

[14] Lindén H, Tetzlaff T, Potjans TC, Pettersen KH, Grün S, Diesmann M et al (2011) Modeling the spatial reach of the LFP. Neuron 72:859–872 22153380 10.1016/j.neuron.2011.11.006

[15] Buzsáki G, Anastassiou CA, Koch C (2012) The origin of extracellular fields and currents — EEG, ECoG, LFP and spikes. Nat Rev Neurosci 13:407–420 22595786 10.1038/nrn3241 PMC4907333

[16] Mazzoni A, Logothetis NK, Panzeri S (2012) The information content of Local Field Potentials: experiments and models. Princ Neural Coding. CRC Press, pp 411–430. Available from: https://arxiv.org/abs/1206.0560v1

[17] Kaplan R, Bush D, Bonnefond M, Bandettini PA, Barnes GR, Doeller CF et al (2014) Medial prefrontal theta phase coupling during spatial memory retrieval. Hippocampus 24:656–665 24497013 10.1002/hipo.22255 PMC4028411

[18] O’Neill PK, Gordon JA, Sigurdsson T (2013) Theta oscillations in the medial prefrontal cortex are modulated by spatial working memory and synchronize with the hippocampus through its ventral subregion. J Neurosci 33:14211–14224 23986255 10.1523/JNEUROSCI.2378-13.2013 PMC3756763

[19] Gehrlach DA, Weiand C, Gaitanos TN, Cho E, Klein AS, Hennrich AA et al (2020) A whole-brain connectivity map of mouse insular cortex. Elife 9:1–78 10.7554/eLife.55585 PMC7538160 32940600

[20] Craig AD (2009) How do you feel — now? The anterior insula and human awareness. Nat Rev Neurosci 10:59–70 19096369 10.1038/nrn2555

[21] Liberati G, Mulders D, Algoet M, van den Broeke EN, Santos SF, Ribeiro Vaz JG et al (2020) Insular responses to transient painful and non-painful thermal and mechanical spinothalamic stimuli recorded using intracerebral EEG. Sci Rep 10:1–15 33339884 10.1038/s41598-020-79371-2 PMC7749115

[22] Baliki MN, Geha PY, Apkarian AV (2009) Parsing pain perception between nociceptive representation and magnitude estimation. J Neurophysiol 101:875–887 19073802 10.1152/jn.91100.2008 PMC3815214

[23] Boivie J (2006) Chapter 48 central post-stroke pain. Handb Clin Neurol Elsevier 81:715–730 10.1016/S0072-9752(06)80052-7 18808870

[24] Hsieh JC, Belfrage M, Stone-Elander S, Hansson P, Ingvar M (1995) Central representation of chronic ongoing neuropathic pain studied by positron emission tomography. Pain 63:225–236 8628589 10.1016/0304-3959(95)00048-W

[25] Murray MG, Gustin SM, Peck CC, Wilcox LS, Henderson LA, Nash GP (2011) Different pain, different brain: thalamic anatomy in neuropathic and non-neuropathic chronic pain syndromes. J Neurosci 31:5956–5964 21508220 10.1523/JNEUROSCI.5980-10.2011 PMC6632967

[26] Harris RE, Sundgren PC, Craig AD, Kirshenbaum E, Sen A, Napadow V et al (2009) Elevated insular glutamate in fibromyalgia is associated with experimental pain. Arthritis Rheum 60:3146–3152 19790053 10.1002/art.24849 PMC2827610

[27] Liberati G, Klöcker A, Algoet M, Mulders D, Safronova MM, Santos SF et al (2018) Gamma-band oscillations preferential for nociception can be recorded in the human insula. Cereb Cortex 28:3650–3664 29028955 10.1093/cercor/bhx237 PMC6366557

[28] Pomares FB, Roy S, Funck T, Feier NA, Thiel A, Fitzcharles MA et al (2020) Upregulation of cortical GABAA receptor concentration in fibromyalgia. Pain 161:74–82 31569142 10.1097/j.pain.0000000000001707 PMC6940028

[29] Mozafarihashjin M, Togha M, Ghorbani Z, Farbod A, Rafiee P, Martami F (2022) Assessment of peripheral biomarkers potentially involved in episodic and chronic migraine: a case-control study with a focus on NGF, BDNF, VEGF, and PGE2. J Headache Pain 23:1–12 34991456 10.1186/s10194-021-01377-6 PMC8903594

[30] Montagnoli C, Tiribuzi R, Crispoltoni L, Pistilli A, Stabile AM, Manfreda F et al (2017) β-NGF and β-NGF receptor upregulation in blood and synovial fluid in osteoarthritis. Biol Chem 398:1045–1054 28253191 10.1515/hsz-2016-0280

[31] Favretti M, Iannuccelli C, Di Franco M (2023) Pain biomarkers in fibromyalgia syndrome: current understanding and future directions. Int J Mol Sci 24:10443 37445618 10.3390/ijms241310443 PMC10341963

[32] Zhang PA, Xu QY, Xue L, Zheng H, Yan J, Xiao Y et al (2017) Neonatal maternal deprivation enhances presynaptic P2X7 receptor transmission in insular cortex in an adult rat model of visceral hypersensitivity. CNS Neurosci Ther 23:145–154 27976523 10.1111/cns.12663 PMC6492683

[33] Zhang PA, Zhu HY, Xu QY, Du WJ, Hu S, Xu GY (2018) Sensitization of P2X3 receptors in insular cortex contributes to visceral pain of adult rats with neonatal maternal deprivation. Mol Pain. 10.1177/1744806918764731 29560791 10.1177/1744806918764731 PMC5865518

[34] Yao X, Li L, Kandhare AD, Mukherjee-Kandhare AA, Bodhankar SL (2020) Attenuation of reserpine-induced fibromyalgia via ROS and serotonergic pathway modulation by fisetin, a plant flavonoid polyphenol. Exp Ther Med 19:1343 32010308 10.3892/etm.2019.8328 PMC6966137

[35] Shafiek MZ, Zaki HF, Mohamed AF, Ibrahim WW (2025) Novel trajectories towards possible effects of semaglutide for amelioration of reserpine-induced fibromyalgia in rats: contribution of cAMP/PKA/p-CREB and M1/M2 microglia polarization. J Neuroimmune Pharmacol 20:1–13. 10.1007/s11481-025-10196-4 10.1007/s11481-025-10196-4 PMC12003577 40240584

[36] El-Sahar AE, Rastanawi AA, El-Yamany MF, Saad MA (2020) Dapagliflozin improves behavioral dysfunction of Huntington’s disease in rats via inhibiting apoptosis-related glycolysis. Life Sci 257:118076 32659371 10.1016/j.lfs.2020.118076

[37] Cordaro M, Siracusa R, D’amico R, Genovese T, Franco G, Marino Y et al (2022) Role of etanercept and infliximab on nociceptive changes induced by the experimental model of Fibromyalgia. Int J Mol Sci 23:6139 35682817 10.3390/ijms23116139 PMC9181785

[38] Ejiri Y, Uta D, Ota H, Mizumura K, Taguchi T (2022) Nociceptive chemical hypersensitivity in the spinal cord of a rat reserpine-induced fibromyalgia model. Neurosci Res 181:87–94 35304863 10.1016/j.neures.2022.03.005

[39] Bradford MM (1976) A rapid and sensitive method for the quantitation of microgram quantities of protein utilizing the principle of protein-dye binding. Anal Biochem Acad Press 72:248–254 10.1016/0003-2697(76)90527-3 942051

[40] Shafiek MZ, Zaki HF, Mohamed AF (2024) New ways to repurpose salmeterol in an animal model of fibromyalgia. Fundam Clin Pharmacol. 10.1111/fcp.13041 39496328 10.1111/fcp.13041

[41] Fornari RV, Wichmann R, Atsak P, Atucha E, Barsegyan A, Beldjoud H et al (2012) Rodent stereotaxic surgery and animal welfare outcome improvements for behavioral neuroscience. J Vis Exp. 10.3791/3528 22314779 10.3791/3528 PMC3353515

[42] Fonoff ET, Dale CS, Pagano RL, Paccola CC, Ballester G, Teixeira MJ et al (2009) Antinociception induced by epidural motor cortex stimulation in Naive conscious rats is mediated by the opioid system. Behav Brain Res 196:63–70 18718490 10.1016/j.bbr.2008.07.027

[43] Silva C, Porter BS, Hillman KL (2021) Stimulation in the rat anterior insula and anterior cingulate during an effortful weightlifting task. Front Neurosci 15:643384 33716659 10.3389/fnins.2021.643384 PMC7952617

[44] Fu B, Wen SN, Wang B, Wang K, Zhang JY, Liu SJ (2018) Acute and chronic pain affects local field potential of the medial prefrontal cortex in different band neural oscillations. Mol Pain. 10.1177/1744806918785686 29902945 10.1177/1744806918785686 PMC6077887

[45] Abo-Zahhad M, Ahmed SM, Abbas SN (2015) A new EEG acquisition protocol for biometric identification using eye blinking signals. Int J Intell Syst Appl 7:48–54

[46] Deuis JR, Dvorakova LS, Vetter I (2017) Methods used to evaluate pain behaviors in rodents. Front Mol Neurosci 10:284 28932184 10.3389/fnmol.2017.00284 PMC5592204

[47] Deuis JR, Dvorakova LS, Vetter I (2017) Methods used to evaluate pain behaviors in rodents. Front Mol Neurosci 10:271711 10.3389/fnmol.2017.00284 PMC5592204 28932184

[48] Leite-Almeida H, Guimarães MR, Cerqueira JJ, Ribeiro-Costa N, Anjos-Martins H, Sousa N et al (2014) Asymmetric c-fos expression in the ventral orbital cortex is associated with impaired reversal learning in a right-sided neuropathy. Mol Pain. 10.1186/1744-8069-10-41 24958202 10.1186/1744-8069-10-41 PMC4106227

[49] Hunt SP, Pini A, Evan G (1987) Induction of c-fos-like protein in spinal cord neurons following sensory stimulation. Nature 328(6131):632–634 3112583 10.1038/328632a0

[50] Wang QP, Zadina JE, Guan JL, Kastin AJ, Funahashi H, Shioda S (2002) Endomorphin-2 immunoreactivity in the cervical dorsal horn of the rat spinal cord at the electron microscopic level. Neuroscience 113:593–605 12150779 10.1016/s0306-4522(02)00153-7

[51] El-Husseini AE-D, Schnell E, Chetkovich DM, Nicoll RA, Bredt DS (2000) PSD-95 involvement in maturation of excitatory synapses. Science 290:1364–1368. 10.1126/science.290.5495.1364 11082065

[52] Zhang X, Li X, Wang H, Xie X, Li Y, Xu X et al (2021) Shank3 contributes to neuropathic pain by facilitating the SNI-dependent increase of HCN2 and the expression of PSD95. Neurosci Res 166:34–41 32454040 10.1016/j.neures.2020.05.010

[53] Chao THH, Chen JH, Yen CT (2018) Plasticity changes in forebrain activity and functional connectivity during neuropathic pain development in rats with sciatic spared nerve injury. Mol Brain 11:1–16 30285801 10.1186/s13041-018-0398-z PMC6167811

[54] Béïque JC, Andrade R (2003) PSD-95 regulates synaptic transmission and plasticity in rat cerebral cortex. J Physiol 546:859–867 12563010 10.1113/jphysiol.2002.031369 PMC2342599

[55] Konan LM, Song H, Pentecost G, Fogwe D, Ndam T, Cui J et al (2019) Multi-focal neuronal ultrastructural abnormalities and synaptic alterations in mice after low-intensity blast exposure. J Neurotrauma 36:2117–2128 30667346 10.1089/neu.2018.6260

[56] Carey L, Lee WH, Gutierrez T, Kulkarni P, Thakur G, Lai Y et al (2017) Small molecule inhibitors of PSD95–nNOS protein–protein interactions suppress formalin-evoked Fos protein expression and nociceptive behavior in rats. Neuroscience 349:303–317 28285942 10.1016/j.neuroscience.2017.02.055 PMC5518314

[57] Henderson LA, Peck CC, Petersen ET, Rae CD, Youssef AM, Reeves JM et al (2013) Chronic pain: lost inhibition? J Neurosci 33:7574–7582 23616562 10.1523/JNEUROSCI.0174-13.2013 PMC6619566

[58] Pyke TL, Osmotherly PG, Baines S (2017) Measuring glutamate levels in the brains of fibromyalgia patients and a potential role for glutamate in the pathophysiology of fibromyalgia symptoms. Clin J Pain 33:944–954 28033157 10.1097/AJP.0000000000000474

[59] Spezia Adachi LN, Quevedo AS, de Souza A, Scarabelot VL, Rozisky JR, de Oliveira C et al (2015) Exogenously induced brain activation regulates neuronal activity by top-down modulation: conceptualized model for electrical brain stimulation. Exp Brain Res 233:1377–1389 25665871 10.1007/s00221-015-4212-1

[60] Hsiao FJ, Wang SJ, Lin YY, Fuh JL, Ko YC, Wang PN et al (2017) Altered insula–default mode network connectivity in fibromyalgia: a resting-state magnetoencephalographic study. J Headache Pain 18:1–10 28831711 10.1186/s10194-017-0799-x PMC5567574

[61] Cook DB, Lange G, Ciccone DS, Liu W-C, Steffener J, Natelson BH (2004) Functional imaging of pain in patients with primary fibromyalgia. J Rheumatol 31:364–378 14760810

[62] Gracely RH, Petzke F, Wolf JM, Clauw DJ (2002) Functional magnetic resonance imaging evidence of augmented pain processing in fibromyalgia. Arthritis Rheum 46:1333–1343 12115241 10.1002/art.10225

[63] Napadow V, LaCount L, Park K, As-Sanie S, Clauw DJ, Harris RE (2010) Intrinsic brain connectivity in fibromyalgia is associated with chronic pain intensity. Arthritis Rheum 62:2545–55 20506181 10.1002/art.27497 PMC2921024

[64] Napadow V, Kim J, Clauw DJ, Harris RE (2012) Brief Report: Decreased intrinsic brain connectivity is associated with reduced clinical pain in fibromyalgia. Arthritis Rheum 64:2398–2403 22294427 10.1002/art.34412 PMC3349799

[65] Flodin P, Martinsen S, Löfgren M, Bileviciute-Ljungar I, Kosek E, Fransson P (2014) Fibromyalgia is associated with decreased connectivity between pain-and sensorimotor brain areas. Brain Connect 4:587–594. 10.1089/brain.2014.0274 24998297 10.1089/brain.2014.0274 PMC4202907

[66] Lutz A, McFarlin DR, Perlman DM, Salomons TV, Davidson RJ (2013) Altered anterior insula activation during anticipation and experience of painful stimuli in expert meditators. Neuroimage 64:538–546. 10.1016/j.neuroimage.2012.09.030 23000783 10.1016/j.neuroimage.2012.09.030 PMC3787201

[67] Fox MD, Snyder AZ, Vincent JL, Corbetta M, Van Essen DC, Raichle ME (2005) The human brain is intrinsically organized into dynamic, anticorrelated functional networks. Proc Natl Acad Sci U S A 102:9673–9678. 10.1073/pnas.0504136102 15976020 10.1073/pnas.0504136102 PMC1157105

[68] Raichle ME, MacLeod AM, Snyder AZ, Powers WJ, Gusnard DA, Shulman GL (2001) A default mode of brain function. Proc Natl Acad Sci 98:676–682 11209064 10.1073/pnas.98.2.676 PMC14647

[69] Farmer MA, Baliki MN, Apkarian AV (2012) A dynamic network perspective of chronic pain. Neurosci Lett 520(2):197–203 22579823 10.1016/j.neulet.2012.05.001 PMC3377811

[70] Jensen KB, Loitoile R, Kosek E, Petzke F, Carville S, Fransson P et al (2012) Patients with fibromyalgia display less functional connectivity in the brain’s pain inhibitory network. Mol Pain 8:1744–8069 10.1186/1744-8069-8-32 PMC3404927 22537768

[71] Cauda F, Sacco K, Duca S, Cocito D, D’Agata F, Geminiani GC et al (2009) Altered resting state in diabetic neuropathic pain. PLoS One 4:e4542 19229326 10.1371/journal.pone.0004542 PMC2638013

[72] Chang LJ, Yarkoni T, Khaw MW, Sanfey AG (2013) Decoding the role of the insula in human cognition: functional parcellation and large-scale reverse inference. Cerebral Cortex 23:739–749 22437053 10.1093/cercor/bhs065 PMC3563343

[73] Kucyi A, Salomons TV, Davis KD (2013) Mind wandering away from pain dynamically engages antinociceptive and default mode brain networks. Proc Natl Acad Sci U S A 110:18692–18697 24167282 10.1073/pnas.1312902110 PMC3832014

[74] Fallon N, Alghamdi J, Chiu Y, Sluming V, Nurmikko T, Stancak A (2013) Structural alterations in brainstem of fibromyalgia syndrome patients correlate with sensitivity to mechanical pressure. NeuroImage Clin 3:163–170 24179860 10.1016/j.nicl.2013.07.011 PMC3791285

[75] Jensen KB, Loitoile R, Kosek E, Petzke F, Carville S, Fransson P, et al. Patients with fibromyalgia display less functional connectivity in the brain’s pain inhibitory network. Mol Pain [Internet]. SAGE PublicationsSage CA: Los Angeles, CA; 2012 [cited 2025 Jan 26];8. Available from: https://journals.sagepub.com/doi/full/10.1186/1744-8069-8-32 10.1186/1744-8069-8-32 PMC3404927 22537768

[76] Ohara S, Crone NE, Weiss N, Lenz FA (2006) Analysis of synchrony demonstrates ‘pain networks’ defined by rapidly switching, task-specific, functional connectivity between pain-related cortical structures. Pain 123:244–253 16563627 10.1016/j.pain.2006.02.012

[77] Liddle EB, Price D, Palaniyappan L, Brookes MJ, Robson SE, Hall EL et al (2016) Abnormal salience signaling in schizophrenia: The role of integrative beta oscillations. Hum Brain Mapp 37:1361–1374 26853904 10.1002/hbm.23107 PMC4790909

[78] Ploner M, Sorg C, Gross J (2017) Brain rhythms of pain. Trends Cogn Sci 21:100–110 28025007 10.1016/j.tics.2016.12.001 PMC5374269

[79] Dimpfel W, Schombert L (2015) Slow gamma activity of local field potentials (LFP) in the freely moving rat relates to movement. J Behav Brain Sci 5:420–429

[80] Trimper JB, Galloway CR, Jones AC, Mandi K, Manns JR (2017) Gamma oscillations in rat hippocampal subregions dentate gyrus, CA3, CA1, and subiculum underlie associative memory encoding. Cell Rep 21(9):2419–2432 29186681 10.1016/j.celrep.2017.10.123 PMC5728687

[81] Mokhothu TM, Tanaka KZ (2021) Characterizing hippocampal oscillatory signatures underlying seizures in temporal lobe epilepsy. Front Behav Neurosci. 10.3389/fnbeh.2021.785328 34899205 10.3389/fnbeh.2021.785328 PMC8656355

[82] Fallon N, Chiu Y, Nurmikko T, Stancak A (2018) Altered theta oscillations in resting EEG of fibromyalgia syndrome patients. Eur J Pain 22:49–57 28758313 10.1002/ejp.1076 PMC5763419

[83] Lim M, Kim JS, Kim DJ, Chung CK (2016) Increased low-and high-frequency oscillatory activity in the prefrontal cortex of fibromyalgia patients. Front Hum Neurosci 10:178762 10.3389/fnhum.2016.00111 PMC4789463 27014041

[84] Stern J, Jeanmonod D, Sarnthein J (2006) Persistent EEG overactivation in the cortical pain matrix of neurogenic pain patients. Neuroimage 31:721–731 16527493 10.1016/j.neuroimage.2005.12.042

[85] Sarnthein J, Stern J, Aufenberg C, Rousson V, Jeanmonod D (2006) Increased EEG power and slowed dominant frequency in patients with neurogenic pain. Brain 129:55–64 16183660 10.1093/brain/awh631

[86] Stern J, Jeanmonod D, Sarnthein J (2006) Persistent EEG overactivation in the cortical pain matrix of neurogenic pain patients. Neuroimage. 10.1016/j.neuroimage.2005.12.042 16527493 10.1016/j.neuroimage.2005.12.042

[87] Walton KD, Dubois M, Llinás RR (2010) Abnormal thalamocortical activity in patients with complex regional pain syndrome (CRPS) type I. Pain 150:41–51 20338687 10.1016/j.pain.2010.02.023

[88] Bjørk MH, Stovner LJ, Engstrøm M, Stjern M, Hagen K, Sand T (2009) Interictal quantitative EEG in migraine: A blinded controlled study. J Headache Pain 10:331–339 19705061 10.1007/s10194-009-0140-4 PMC3452093

[89] Knyazev GG (2011) Cross-frequency coupling of brain oscillations: an impact of state anxiety. Int J Psychophysiol 80:236–245 21458502 10.1016/j.ijpsycho.2011.03.013

[90] Styliadis C, Kartsidis P, Paraskevopoulos E, Ioannides AA, Bamidis PD (2015) Neuroplastic effects of combined computerized physical and cognitive training in elderly individuals at risk for dementia: an eLORETA controlled study on resting states. Neural Plast 2015:172192 25945260 10.1155/2015/172192 PMC4405298

[91] Gram M, Graversen C, Olesen SS, Drewes AM (2015) Dynamic spectral indices of the electroencephalogram provide new insights into tonic pain. Clin Neurophysiol 126:763–771 25213351 10.1016/j.clinph.2014.07.027

[92] Craig A, Tran Y, Wijesuriya N, Nguyen H (2012) Regional brain wave activity changes associated with fatigue. Psychophysiology 49:574–582 22324302 10.1111/j.1469-8986.2011.01329.x

